# Clinical characterization of a hypersensitivity mixed bacterial and fungal dermatitis in a translational model of porcine NASH

**DOI:** 10.3389/fcimb.2023.1277045

**Published:** 2024-01-24

**Authors:** Philipp Felgendreff, Josephine M. Lawrence, Seyed M. Hosseiniasl, Julie F. Jacobs, Bruce P. Amiot, Lisa Felgendreff, Anna Minshew, Ahmer Sultan, Boyukkhanim Ahmadzada, Michael C. Rahe, Scott L. Nyberg

**Affiliations:** ^1^ Department of Surgery, Mayo Clinic, Rochester, MN, United States; ^2^ Department of General, Visceral, and Transplantation Surgery, Hannover Medical School, Hannover, Germany; ^3^ Department of Comparative Medicine, Mayo Clinic, Rochester, MN, United States; ^4^ Center for Empirical Research in Economics and Behavioral Sciences, Media and Communication Science, University of Erfurt, Erfurt, Germany; ^5^ Veterinary Diagnostic and Production Animal Medicine, Iowa State University, Ames, IA, United States; ^6^ Population Health and Pathobiology, North Carolina State University, Raleigh, NC, United States; ^7^ William J. von Liebig Center for Transplantation and Clinical Regeneration, Mayo Clinic, Rochester, MN, United States

**Keywords:** NASH, skin evaluation system, large animal model, microbiome, dermatitis

## Abstract

**Introduction:**

The development of animal models of chronic liver disease via diet modification is a promising avenue for translational research but can lead to unexpected side effects that impact model adoption. While these side effects are well characterized in rodent models of nonalcoholic steatohepatitis (NASH), limited knowledge of these effects exists for novel porcine models of NASH. To close this gap, the present study investigates the side effects of diet-based NASH induction in pigs, with a systematic analysis of the pathologic mechanisms underlying dermatitis development and evaluation of treatment approaches.

**Method:**

Twelve pigs (10 large domestic pigs, 2 Goettingen minipigs) were fed a methionine- and choline-deficient, high-fat diet for 8 weeks to induce NASH. A retrospective review of each animal’s clinical record was performed to identify the side effects of the diet. Following the identification of diet-associated dermatitis, severity was judged by using a novel gradation system that characterized the individual lesions and body regions resulting in a cumulative evaluation. In addition to this clinical assessment, the etiology of the dermatitis was investigated via histopathologic and microbiologic testing. Furthermore, the success of prophylactic and therapeutic treatment approaches was evaluated by considering dermatitis development and clinical course.

**Results:**

All study animals demonstrated unexpected side effects of the methionine- and choline-deficient, high fat diet. In addition to marked dermatitis, study pigs showed impaired weight gain and developed steatorrhea and anemia. Based on the skin gradation system, five animals developed severe dermatitis, four animals moderate dermatitis, and three animals mild diet-associated dermatitis. Histological and microbiological evaluation of the affected skin showed signs of a hypersensitivity reaction with secondary infection by bacteria and fungi. The analysis showed that preemptive bathing extended the lesion-free duration by nearly 20 days. Furthermore, bathing in combination with a targeted antibiotic treatment represented a helpful treatment approach for diet-associated dermatitis.

**Conclusion:**

The provision of a methionine- and choline-deficient, high fat diet represents an effective approach for inducing NASH liver disease in pigs but predisposes study animals to multiple side effects. These side effects are universal to animals on study but can be adequately managed and do not represent a significant limitation of this model.

## 1 Introduction

Translational research of innovative technologies and new experimental approaches requires animal models that are analogous to humans. Basic research and the early developmental stages of therapeutic interventions rely primarily on rodent models, but large animal models are essential in the approval process to evaluate the efficacy, durability, dose response, degradation, and safety of advanced therapeutic medicinal products (ATMPs). The US Federal Food and Drug Administration (FDA), European Medicines Agency (EMA), and the International Society for Stem Cell Research (ISSCR) recommend the use of large animal models to evaluate therapeutic interventions prior to their use in humans, as this is critical to ensuring the translatability of the model.

The choice of an appropriate large animal model is essential to obtaining meaningful results and fulfilling the study aim. Furthermore, the selection of a model is crucial for the design of the study and both biological and practical considerations must be weighed. Due to their anatomic and physiological similarities with humans, as well as their adaptability to a lab setting, fecundity, and variety of breeds, swine are a frequently chosen animal for translational research. Research use of swine is varied, with widespread development of swine models of hepatobiliary disease (Swindle et al., 2012). While pig liver models are routinely used for acute or short-term studies evaluating surgical or drug-based interventions, an increasing number of chronic studies are being conducted with models of diet-induced chronic liver failure (Schumacher-Petersen et al., 2019; Pedersen et al., 2020).

Disease induction via diet manipulation has resulted in the development of multiple swine disease models. Alteration of the diet’s caloric content or nutrition profile has resulted in swine models of obesity, malnutrition, diabetes, and atherosclerosis (Xi et al., 2004; Jové et al., 2020; Rodríguez et al., 2020; Lacaze et al., 2022). Research using these models focuses on the characterization of the model in comparison to humans, the potential evaluation for surgical and medication-based therapeutic interventions, and the associated limitations of these approaches. Successful implementation of these models has encouraged the development of further diet-induced disease models to test therapeutic interventions. However, experiences in porcine models to test innovative therapeutic strategies in liver disease are still very limited.

Provision of a methionine- and choline-deficient, high-fat diet is a promising approach to inducing nonalcoholic steatohepatitis (NASH) in pigs (Schumacher-Petersen et al., 2019; Pedersen et al., 2020; Herrera-Marcos et al., 2022). This approach is well-established in rodents and has been transferred to pigs, representing an interesting starting point for various translational research purposes. The opportunity to evaluate new surgical- and drug-based approaches designed in rodents and shifted to animals that more closely model humans opens new opportunities to transfer knowledge from bench to bedside. However, the development of such chronic liver failure models in pigs is always associated with questions regarding general study-related animal welfare and the potential of diet-associated side effects.

The side effects of inducing NASH liver disease via a methionine- and choline-deficient, high fat diet- are well known in rodents. In these animals, reduced appetite in combination with weight loss and dysregulation of the reproduction cycle leading to general welfare concerns are established, limiting the long-term use of this model (Matsumoto et al., 2013; Kroh et al., 2020). These welfare concerns are further compounded by the risk of incisor overgrowth and altered coat conditions in these animals, impacting their clinical care and husbandry needs. The presence of these side effects in rodent models of NASH suggests that side effects are not unlikely in swine models of NASH.

As a novel porcine model of NASH, the side effects of dietary manipulation in pigs are less well known than in rodents, leaving the etiology and treatment approaches for these effects unstudied. Disease induction via diet manipulation has demonstrated side effects in other swine models of disease, but the currently available clinical data related to the impact of NASH diet in pigs appears to be incomplete and can lead to unreasonable speculations for studies involving pigs (Schumacher-Petersen et al., 2019; Pedersen et al., 2020; Viuff et al., 2020). Understanding and handling of dietary-related side effects are vital for study staff when selecting animal models, veterinary staff when implementing monitoring plans, and animal research committees when evaluating proposed protocols. The lack of published research concerning side effects is further compounded by a similar lack of scoring systems to adequately evaluate those animals that develop disease. Although established scoring systems for evaluating body condition are present, monitoring systems for assessing expected dermatologic dietary side effects are currently underrepresented for pigs (Renggaman et al., 2015; Norring et al., 2019).

To close this gap of knowledge, this study aims to characterize unanticipated side effects in a chronic, diet-induced model of NASH in swine. Special attention will be paid to dermatologic manifestations over the NASH induction period, including the gradation of the skin disease, histological and microbiological characteristics of the lesions, and first limited clinical experiences of developed treatment plans.

## 2 Method

### 2.1 Study design

**Figure 1:**
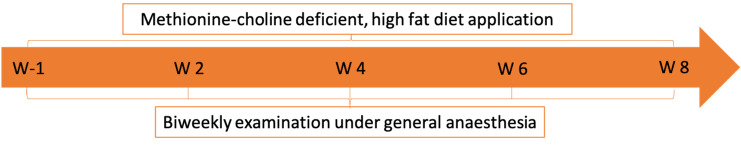
NASH induction-study course including the time points of clinical examination (W-1: 1 week prior to the start of the methionine- and choline-deficient high fat diet, W 2: 2 weeks after diet start, W 4: 4 weeks after diet start, W 6: 6 weeks after diet start, W 8: 8 weeks after diet start).

The side effects of NASH induction by feeding a methionine- and choline-deficient, high fat diet over 8 weeks (**Figure 1**) were identified by retrospective analysis of pig health status as documented in individual clinical records. Clinical records were maintained by veterinarians, veterinary technicians, and investigators. All animals were assessed daily, and changes in health status, behavior, and stool consistency were noted in the clinical record. Additionally, animals were weighed weekly and closely examined, including photo documentation of skin lesions, on a biweekly basis during general anesthesia. During these sedation events, histological samples of the skin, complete blood counts, swab samples of skin, and blood culture samples were taken. Diet-induced dermatitis was judged with a three-step gradation approach: (1) Evaluation of the individual lesions based on the intensity of the dermatitis; (2) Evaluation of the distribution of the lesions on basis of each anatomic region of the pig; (3) Cumulative evaluation of the skin condition of the entire animal by combining the individual judgment of the lesions and their distribution over the animal surface.

### 2.2 Animals and study manipulation

#### 2.2.1 Animals

The design of the study and the involved animal work was approved by the Institutional Animal Care and Use Committee (IACUC) of Mayo Clinic. This study was performed with wild-type female domestic pigs and Gottingen mini-pigs. Two Gottingen mini-pigs were purchased from Marshall BioService, NY and tested negative for porcine epidemic diarrhea virus, porcine circovirus 2 (PCV2), influenza A virus, porcine parvovirus-1, porcine reproductive and respiratory syndrome virus (PRRSV), transmissible gastroenteritis virus, pseudorabies virus, *Actinobacillus pleuropneuomoniae*, *Brucella suis*/*abortus*, *Erysipelothrix rhusiopathiae*, *Glaesserella parasuis*, *Lawsonia intracellularis*, and *Leptospira pomona*, *icterohaemorrhagiae*, *canicola*, *hardjo*, and *grippotyphosa* prior their arrival.

Ten domestic pigs were purchased from a local commercial operator (Manthei Hog Farm, Elk River, MN). These animals were negative for PRRSV, *Mycoplasma hyopneumoniae*, *Brucella suis/abortus*, and pseudorabies and were vaccinated against PCV2, *Lawsonia intracellularis*, and *Mycoplasma hyosynoviae* and *hyorhinis* prior their shipment. After arrival, all animals were vaccinated against *Erysipelothrix rhusiopathiae* (RespiSure-One/ER Bac Plus, Zoetis, Parsippany-Troy Hills Township, New Jersey) ¸ *Mycoplasma hyopneumoniae* (RespiSure-One/ER Bac Plus, Zoetis, New Jersey), and *Glaesserella parasuis* (Para Sheild, Elanco, Indiana) with booster vaccination two weeks later. Gottingen mini-pigs were additionally vaccinated against PCV2 (Circumvent PCV M, Intervet, New Jersey).

#### 2.2.2 Housing

Pigs were housed and cared for in compliance with the Guide for the Care and Use of Laboratory Animals, 8^th^ edition, in an AAALAC-accredited facility. Animals were singly housed in pens measuring 48in (122cm) x 72in (183cm) with open slots between pens to allow socialization between animals. Single housing was required in order to monitor animal-specific diet intake. Pigs were provided ad libitum water through an automatic watering system. Animals were maintained at a 12:12 light: dark cycle with relative humidity and room temperature between 30% and 70% and 61-81° F (16°C-28°C), respectively. Supplemental heating lamps were placed if needed. Enrichment was provided through toys switched weekly, food treats, visual and auditory contact with conspecifics, and regular positive interaction with humans.

#### 2.2.3 Main study – development of NASH liver disease

The main study of NASH liver development in pigs was performed by an interdisciplinary team including hepatobiliary surgeons, veterinarians, hepatologists, and a pathologist. A commercially available methionine- and choline-deficient diet (TD.90262, Teklad, WI) was fed over a period of 8 weeks to induce NASH liver disease. The diet was stored at +4˚C and fed twice daily in animal specific portions (portion size (g): 5%-7% of the current bodyweight(g)). Banana flavoring and methionine-and choline-deficient marshmallows were added to the diet to encourage ingestion and provided individually as enrichment treats. All animals were anesthetized prior to diet initiation and biweekly after the start of the feeding period as part of the main NASH development study (blood draws, liver biopsies) to confirm NASH development and to monitor potential side effects of the diet (changes in the health status, performing skin biopsies, taking skin swab tests, draw blood culture samples).

### 2.3 Dermatitis evaluation

#### 2.3.1 Gradation of diet-induced dermatitis

Grading of each animal’s skin was performed via retrospective analysis of clinical records, including written descriptions, pictures, and systematic examinations under general anesthesia. A single reviewer developed the scoring system and evaluated all animals. The skin evaluation was broken into eight, week-long evaluation periods. A stable skin condition was assumed if no changes in skin condition was mentioned in the medical records. The skin was judged according to the following three step approach:

1. Assessment of the individual lesions according to the development of crust and scabs, signs of inflammation, and evidence of wound healing.
2. Presence or absence of lesions at each anatomic region.
3. Cumulative evaluation of the animal’s dermatitis via incorporation of the individual assessments at each respective anatomic regions, allowing a comprehensive assessment of the pig skin condition.

#### 2.3.2 Judgment of individual lesions

The judgment of individual lesions was based on severity as characterized by the following factors:

1. Presence of crusts and scabs as signs of wound effusion.
2. Presence of erythematic margins as signs of acute inflammation.
3. Presence of re-epithelialization associated with skin discoloration as a sign of wound healing.

Based on these aspects, each lesion was assigned a specific gradation as follows (**Table 1**):

**Table 1:** Overview of the lesion-specific gradation system with subsequent points to evaluate the dermatitis associated with the application of a methionine-, choline-deficient, high fat diet.

Skin with acute dermatitis lesions (3 points)	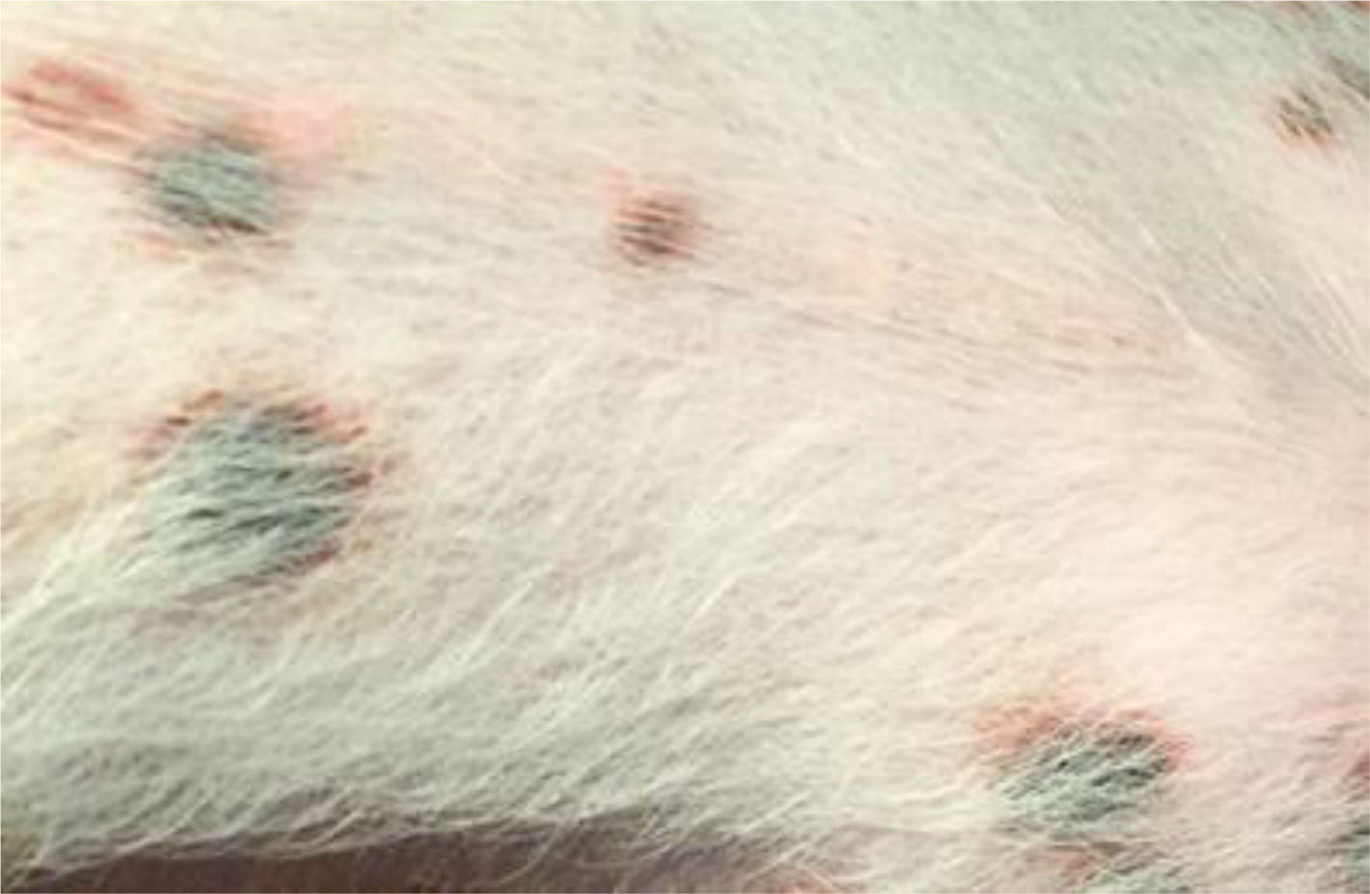 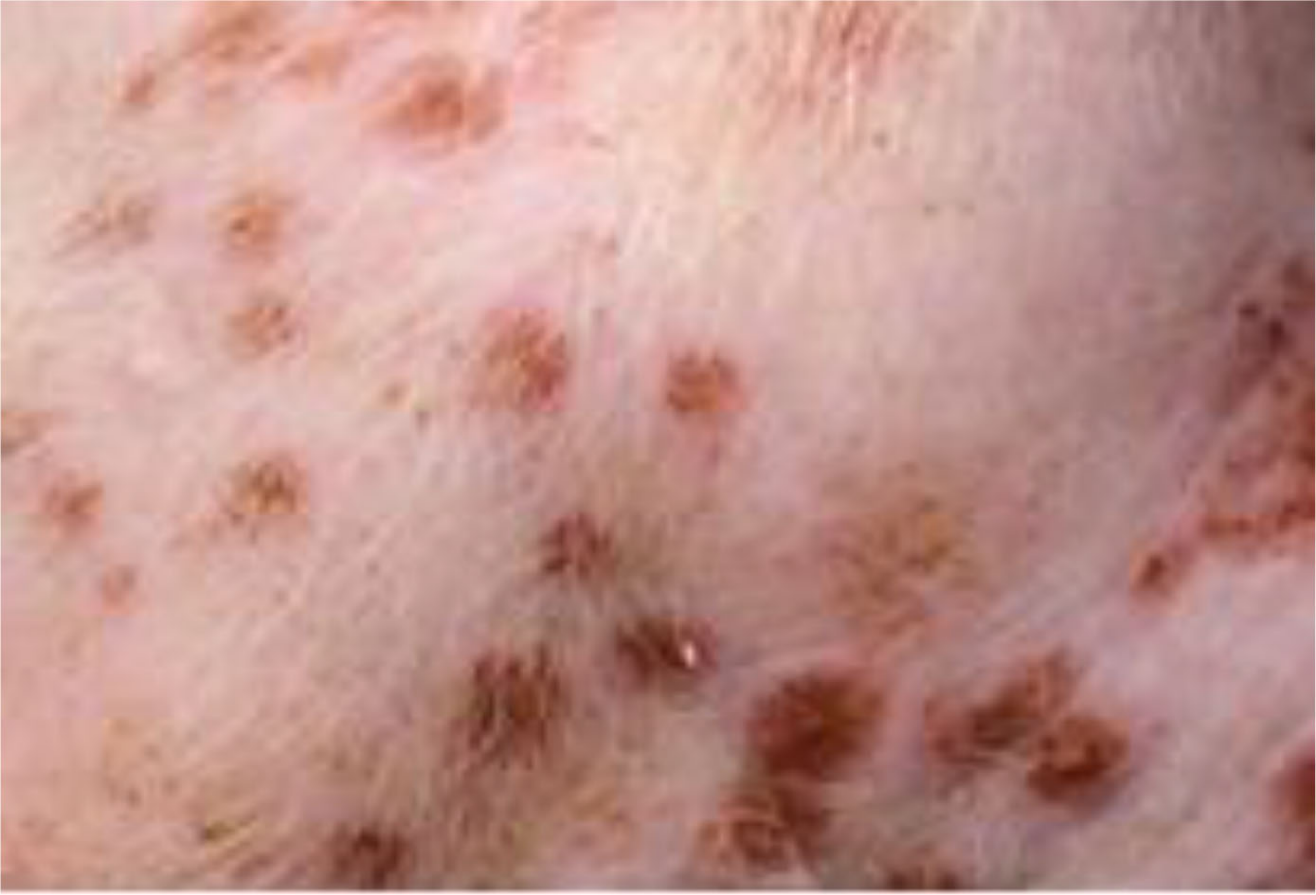	Diet-induced dermatitis with erythemic, encircled, scabbed or pustular lesions. No evidence of re-epithelization.
Skin with latent dermatitis lesions (2 points)	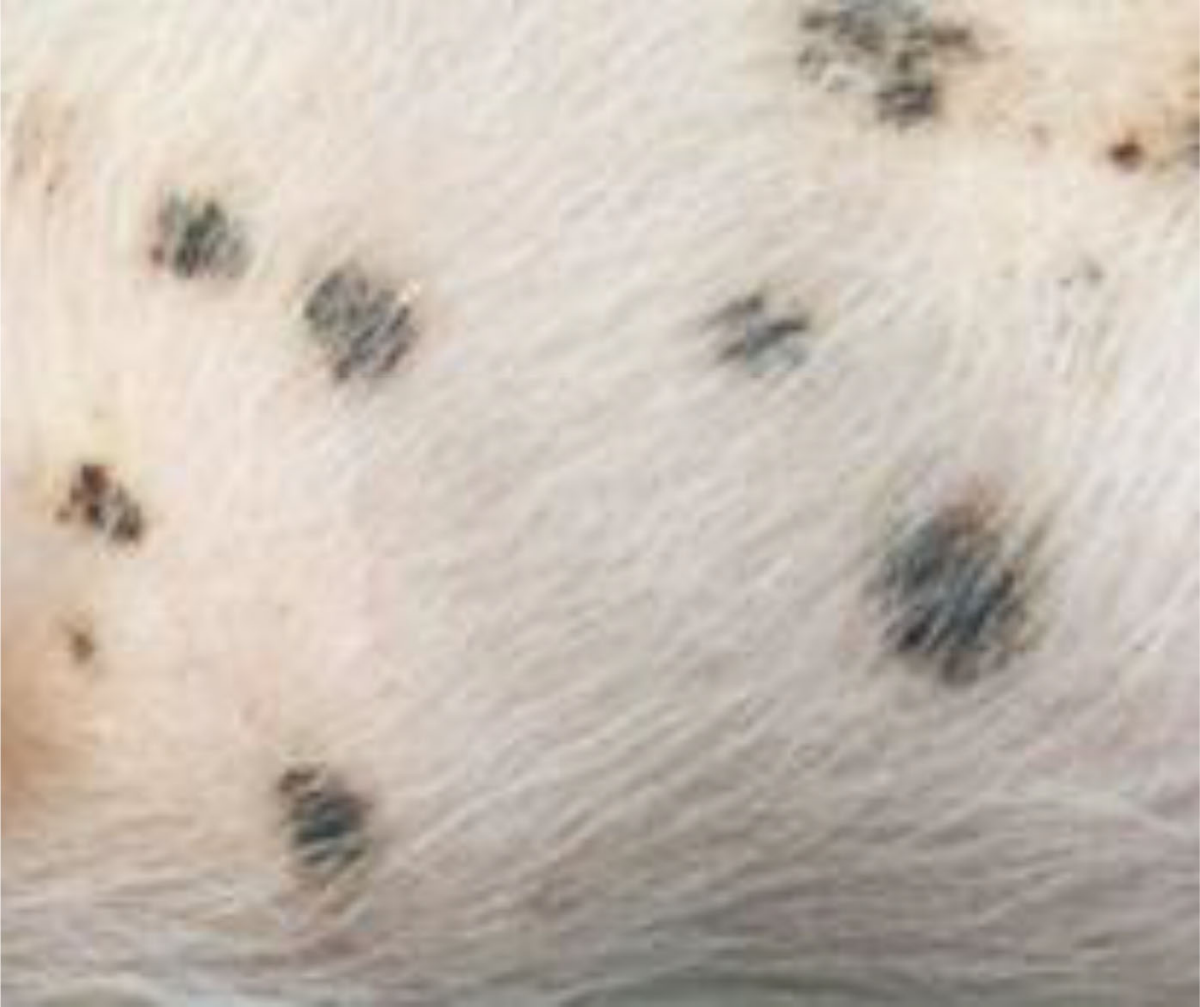	Diet-induced dermatitis with scabbed or pustular lesions, no evidence of erythemic margins or re-epithelization.
Skin with dermatitis lesions in remission (1 points)	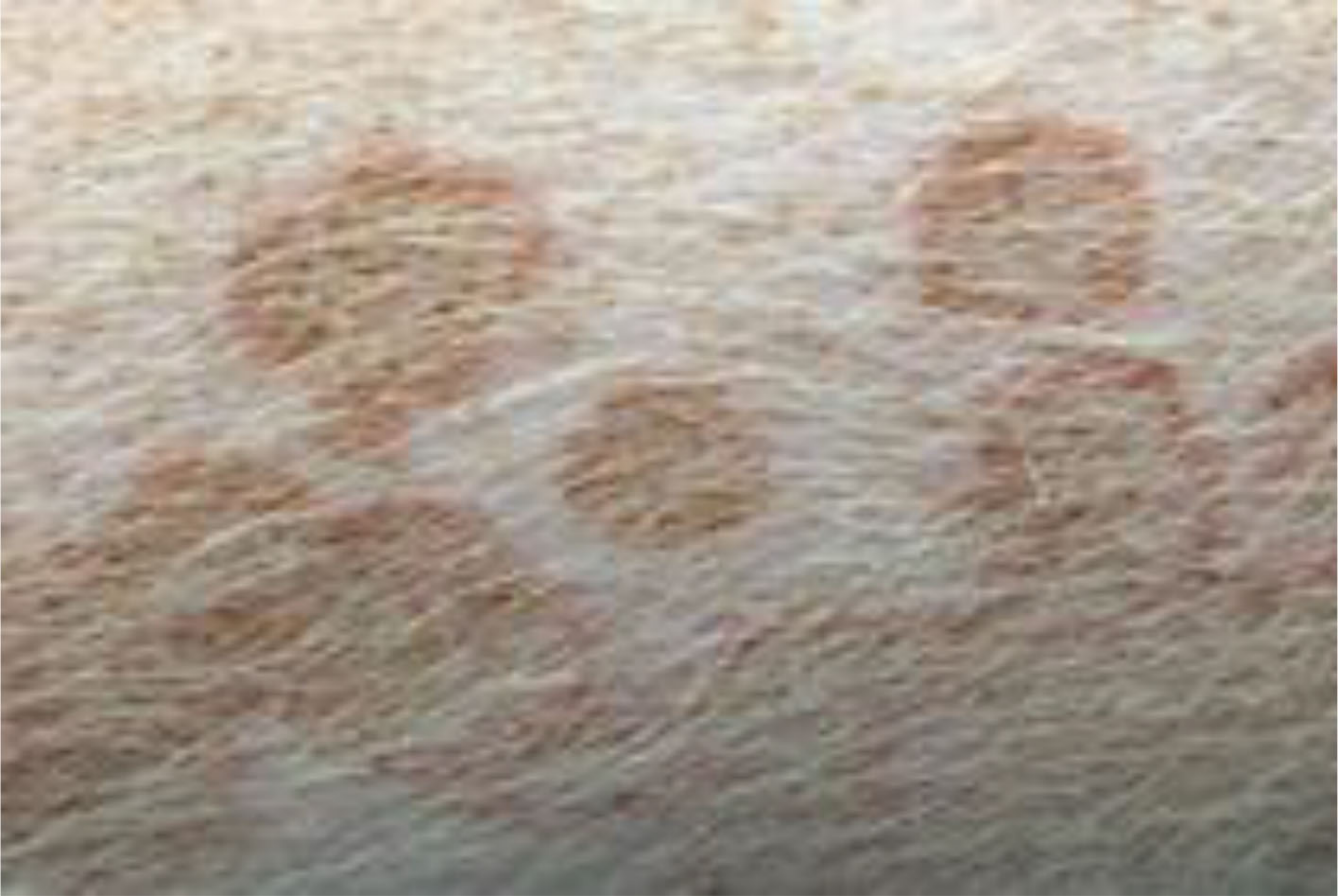	Diet-induced dermatitis with evidence of re-epithelization and associated skin discoloration. No evidence for erythemic, encircled, scabbed or pustular lesions
Skin without dermatitis lesions (0 points)	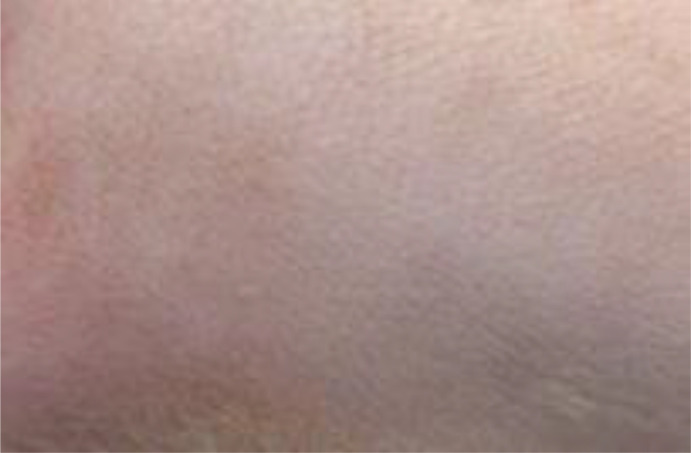	Normal pig skin without erythemic encircled scabbed or pustular lesions, no evidence of re-epithelization.

#### 2.3.3 Judgment of anatomic regions

**Figure 2:**
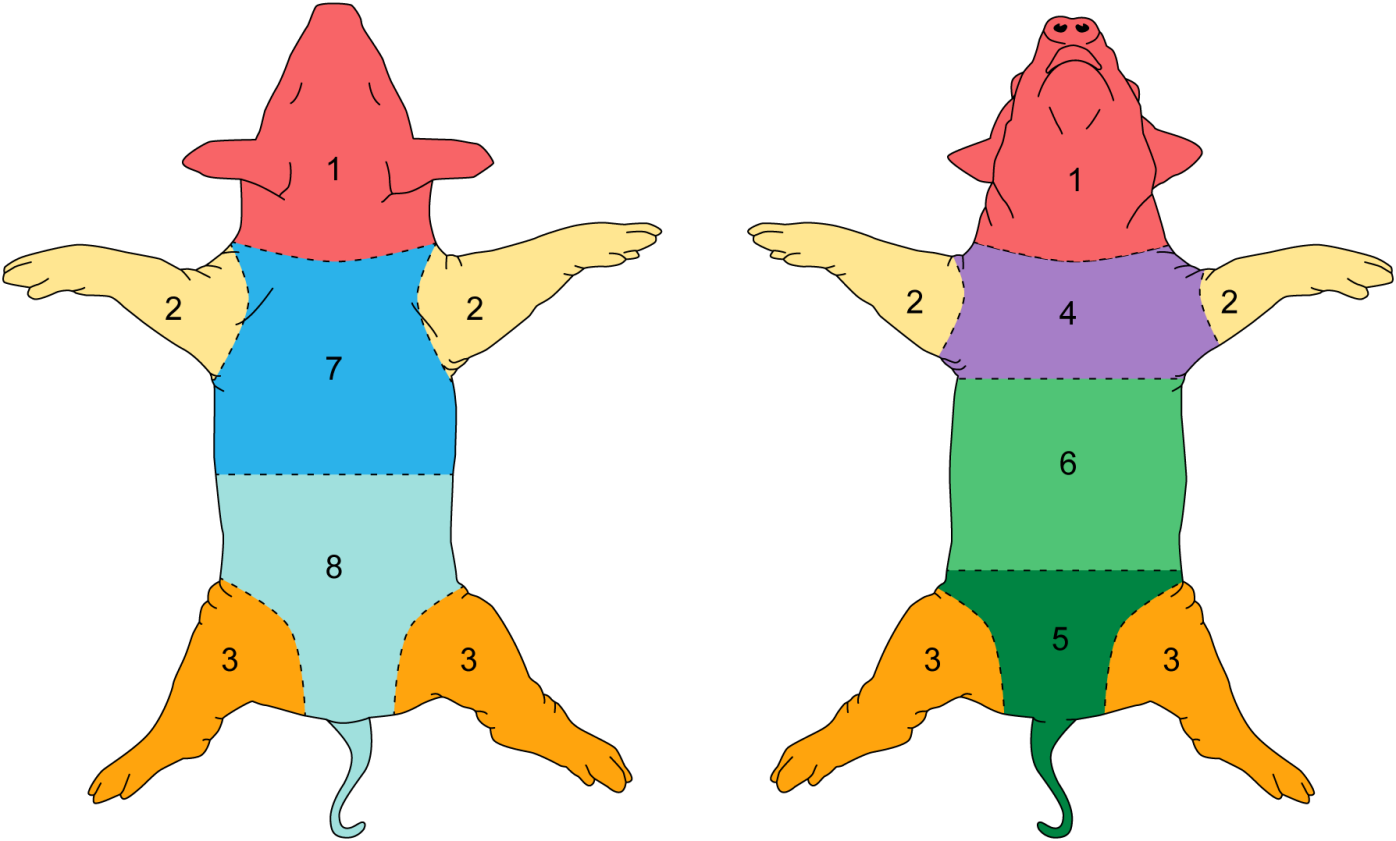
Individual skin regions of the pig.

To grade the entire skin surface of each animal systematically, the skin was divided in eight bilateral areas. For affected regions the most severe lesion in each region was used to grade the entire region. **Figure 2** shows the division of anatomic regions.

#### 2.3.4 Cumulative skin gradation

A cumulative score (C) for the entire individual animal was determined by incorporating the individual lesion score at each anatomic region (s), then incorporating all anatomic region scores into a single value by using the following formula:


$$\text{C = }\sum_{i=1}^{8}S_{\text{i}}$$

A single cumulative score was assigned per week, for a total of eight weekly scores. To classify the severity of the diet-induced dermatitis, three groups were defined based on the highest score achieved over the eight-week feeding period. Pigs were placed into one of the following groups:

- Severe NASH dermatitis = Skin score between 24 points and 17 points
- Moderate NASH dermatitis = Skin score between 9 points and 16 points
- Mild NASH dermatitis = Skin score between 1 point and 8 points

#### 2.3.5 Dermatitis treatment evaluation

Dermatitis was managed via topical and systemic treatment. Dermatitis was initially topically managed in a curative setting via bathing affected animals with 4% chlorohexidine shampoo daily to weekly, at veterinary discretion, based on lesion severity. Following the initial set of four study animals, later animals on study were bathed with 4% chlorohexidine shampoo (ChlorHex 2X, Vedco, St. Joseph, Missouri) weekly following diet initiation in a preemptive approach. Days to lesion development for pre-emptively bathed and retroactively pigs were evaluated.

Two animals with severe, rapidly progressive lesions that were resistant to bathing alone were additionally treated with systemic antibiotic therapy at veterinary discretion (Enrofloxacin 7.5 mg/kg, 90 mg, once daily and amoxicillin/clavulanic acid 20 mg/kg, 200-250 mg, twice daily). The choice of antibiotic therapy was initially empirical and transitioned to targeted therapy based on culture and sensitivity results. Changes in skin grading following antibiotic treatment was evaluated for the pigs that received antibiotic therapy.

### 2.4 Diagnostic sampling

Diagnostic sampling was performed prior to diet initiation (W-1) and each second week (W2, W4, W6, W8) during the feeding period. Animals were maintained under general anesthesia (induction: 5 mg/kg Telazol, 2 mg/kg Xylazine; maintenance: inhaled isoflurane 1%−2%) and vital signs were recorded every 15 minutes to ensure sufficient monitoring of the animal during the sedation period.

#### 2.4.1 Complete blood count, clinical chemistry and blood culture sampling

Under general anesthesia, complete blood counts (CBC), clinical chemistry (CC) (urea, total bilirubin and total protein) and blood culture samples were collected via aseptic, ultrasound-guided femoral venipuncture. CBC and CC samples were taken prior (W-1) and during the regularly planned diagnostic sampling time points (W2, W4, W6, W8). CBC analysis was performed with Abaxis HM5 VetScan Hematology analyzer (Axonia medical, Singapore). The CC samples were performed using Piccolo Xpress comprehensive metabolic panel (Abaxis, Union City, CA). Blood culture samples were collected from two animals prior and in the fourth week of the feeding period following dermatitis development. The blood culture samples were cultured under aerobic and anaerobic conditions at a commercial laboratory (IDEXX BioAnalytics, Columbia, MO).

#### 2.4.2 Culture and sensitivity

Culture and sensitivity samples were taken by swabbing underneath affected, scabbed sites. Swabs were sent to a veterinary diagnostic laboratory for aerobic and anaerobic bacterial culture, fungal culture, and antibiotic susceptibility (Iowa State University, Veterinary Diagnostic Laboratory, Ames, IA).

Bacteriology setup was done for routine culture. Swabs were cultured aerobically and anaerobically at 35°C for 2 days on 5% sheep blood agar, Tergitol-7 agar, and HE agar (Remel/ThermoFischer, Lenexa, KS). Culture restreaks were performed on 5% sheep blood agar until pure cultures were grown. Identification of pure cultures was performed using MALDI-TOF MS (Brunker MBT). All growth was reported and tested for antimicrobial susceptibility using veterinary CLSI guidelines for test performance and interpretation of minimum inhibitory concentration results. Sensitivity interpretations were extrapolated from other hosts and closest organisms as appropriate by the manufacturers’ AST software (Sensititre AST System, ThermoFischer, Waltham, MA).

Fungal culture was performed on mycotic Agar, BHI agar, and IMA agar incubated at 35°C and SD agar incubated at 25°C and 35°C for up to 4 weeks under aerobic conditions. Fungal identification was performed through the evaluation of phenotypic characteristics (growth characteristics and morphology) and MALDI-TOF MS.

#### 2.4.3 Histopathology

Under general anesthesia, 4 and 7 mm punch skin biopsies were taken from affected and unaffected skin regions. The samples were fixed in 5% buffered formalin for 48h and cut (5μm thick) after paraffin embedding. The sections were subjected to routine hematoxylin/eosin as well as Gram and Grocott’s methenamine silver (GMS) staining following a standard protocol. Additional immunohistochemical (IHC) staining was performed on FFPE tissues sectioned at 4 µm and placed on Superfrost plus (VWR) slides. Three antibodies were used at the stated dilutions with noted deviations from the previously described Ventana Discovery Ultra protocol (Rahe et al., 2022).

1) CD3 (T cells) and CD20 (B cells)
  a) CD3 - A045201-2 (Agilent technologies) at a 1:200 dilution
  b) CD20 – Polyclonal (Biocare Medical) ACR 3004 at a 1:200 dilution
  c) Step 2.) Cell conditioning 1 for 48 min at 100°C
  d) Step 5.) Anti-rabbit HQ application and incubation
  e) Step 6.) Detection with anti-HQ HRP
2) Iba1 (macrophages)
  a) Ab5076 (Abcam) at a 1:500 dilution
  b) Step 2.) Cell conditioning 1 for 48 min at 100°C
  c) Step 5.) Discovery OmniMap anti-goat and antibody block

All sections were digitalized using a MoticEasyScan Pro slide scanner (Motic Digital pathology, San Francisco, California). The diagnostic evaluation of the samples was performed by two board certificated veterinary pathologists at the Iowa State University Veterinary Diagnostic Laboratory (ISU VDL) a veterinary diagnostic laboratory (Veterinary Diagnostic Laboratory, Ames, IA).

### 2.5 Statistical analysis

Group comparisons regarding the skin gradation system were performed using R (Tierney, 2012) (R-Core Team) and the package compareGroups (Subirana et al., 2014). The Shaphiro Wilk test was used to test whether the continuous variables can be considered as normal distributed or not. In the case of normal distribution, the mean and the standard derivation (round brackets) were reported. Group differences were tested with an analysis of variance (ANOVA). In the case of non-normal distributed continuous variables, the median and the first and third quartile (square brackets) were indicated, and the groups were compared using the Kruskall-Wallis test (Subirana et al., 2014).

## 3 Results

### 3.1 Animal data

The main study to develop NASH in pigs was performed by the Department of Surgery at Mayo Clinic, Rochester, MN. A total of 12 female pigs were included on study: 2 Gottingen mini-pigs and 10 domestic pigs. The average starting weight for the domestic and Gottingen minipigs was 12.1kg ( ± 1.17) and 5.8kg ( ± 0.28), respectively (**Table 2**). All animals were apparently healthy on arrival with no signs of systemic or skin related disease prior to study initiation. Animals were acclimated for a minimum of five days on arrival to the facility in accordance with Mayo Clinic IACUC policy and were fed a commercial swine diet until study start (Purina, Minnesota). The 8-week feeding period was completed in all animals. No animal was excluded from the study due to unexpected side effects of the diet or other non-diet related medical reasons.

**Table 2:** General characteristics of all included animals.

	Domestic	Gottingen
Total number	10	2
Sex (male/female)	0/10	0/2
Average body weight ( ± SD) (kg)	12.1 ( ± 1.17)	5.8 ( ± 0.28)
Days on NASH diet	56	56

SD, standard deviation.

### 3.2 Side effects of diet

All animals enrolled in the NASH development study completed the study course as planned. The retrospective analysis of the animal specific clinical records identified four major side effects associated with the study diet.

The analysis identified four major side effects associated with the study diet. However, the severity and clinical manifestation of these side effects was different in all animals. All animals showed an impaired growth rate indicated by a failure to gain signficant weight during the feeding period (**Supplementary Figure 1**). Furthermore, the included animals developed loose, gray to tan stool along the feeding period and the hemoglobin values in all animals were reduced compared to reference levels, indicating impaired hematopoiesis during the study course. (**Supplementary Figure 2**). The largest differences in the clinical expression of side effects were noticed in the development of diet-associated dermatitis. All included animals developed dermatitis lesions associated with the diet application, but the period until the first lesions appeared as well as the severity of disease differed markedly between animals.

#### 3.2.1 Evaluation of dermatitis via skin gradation

**Figure 3:**
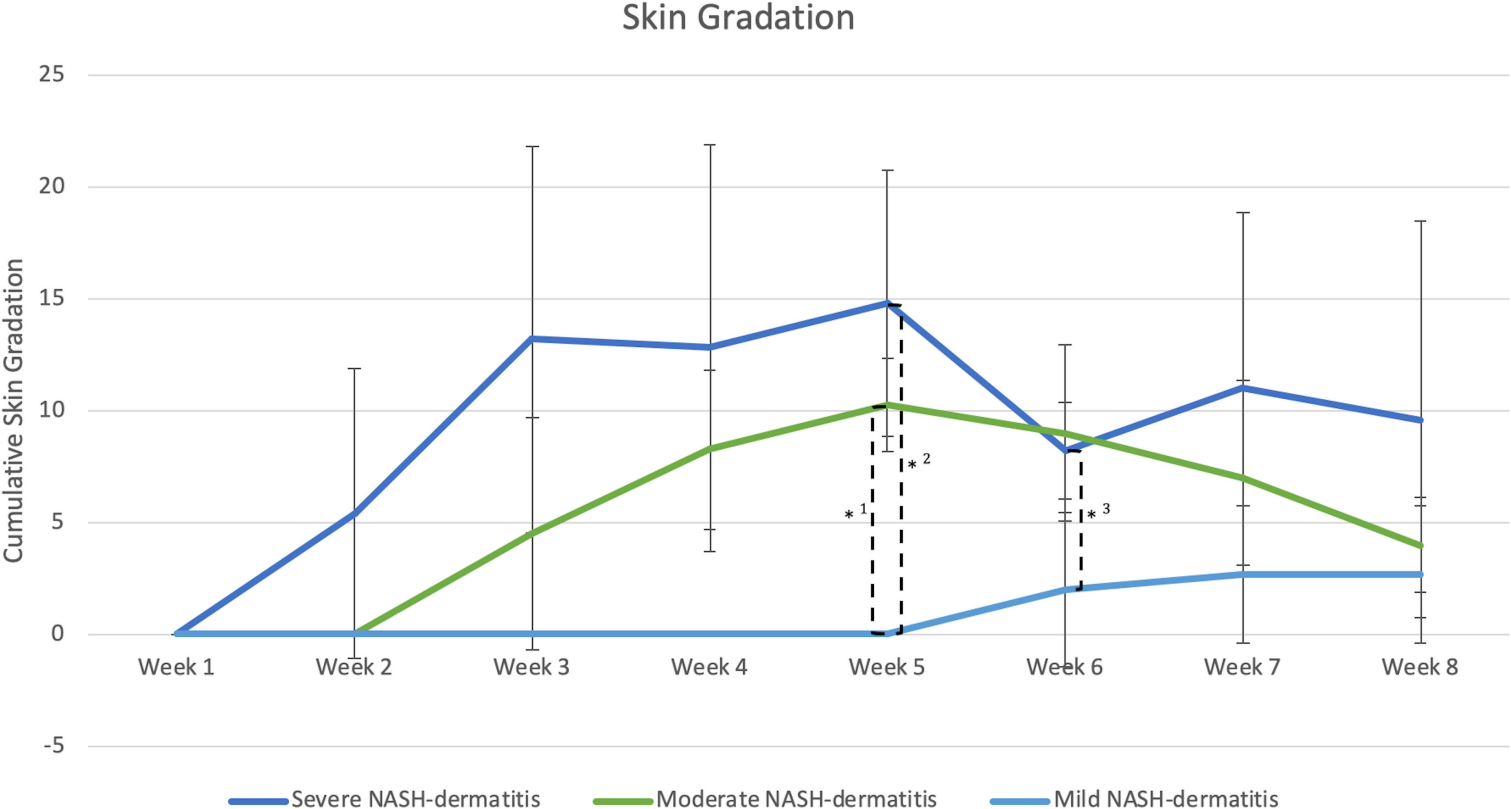
(n=3) =Skin score between 1 point and 8 points, *1: Significant different skin gradation comparing moderate and mild NASH dermatitis at week 5 of the study course (p-value: 0.02); *2: Significant different skin gradation comparing severe and mild NASH dermatitis at week 5 of the study course (p-value: 0.01); *3: Significant different skin gradation comparing severe and mild NASH dermatitis at week 6 of the study course (p-value: 0.049).

The evaluation of dermatitis was accomplished by judging eight anatomic regions over the feeding period of eight weeks, a total of 758 individual regions in twelve animals, considering the four potential skin gradations (acute dermatitis, latent dermatitis, dermatitis in remission, normal skin) (**Figure 3**).

All included animals had normal skin without any evidence of dermatitis prior to and in the first week of the feeding period. According to the gradation system and assigned mild, moderate, and severe scoring categories, five animals were classified with severe dermatitis, four with a moderate dermatitis, and three with a mild dermatitis. The rate of lesion progression and peak score differed between severity categories. In the severe group, the diet-induced dermatitis score rose rapidly but stabilized in week 3 of the study course with a peak at week 5 (average score: 14.8 ( ± 5.93)). The progress in the moderate and mild group was significantly slower. The peak in the moderate (average score 10.25 ( ± 2.06)) and mild (average score: 2.6 ( ± 3.06)) group was in week 5 and 7, respectively. Development of lesions in the mild group was significantly delayed, with pigs in the mild group showing the first signs of dermatitis in week 6 of the study. The statistical analysis of the data revealed significant differences in the skin score between the three study groups at certain time points along the course of the study. At week 5, the animals in the moderate and mild (p=0.02) as well as the severe and mild (p=0.01) groups showed a statistical difference in skin score. In addition, the skin score of the animals with a severe skin score was significantly higher in week 6 of the study (p=0.049) than in the animals with a moderate skin score (**Figure 3**). Further analysis of the data identified anatomic regions which were more likely to be affected based on the severity of the lesion. By comparing the animals in the moderate and mild groups, region 3, representing the hind limbs, was significantly more often affected than pigs in the mild group. Regions 5, 6, and 7, representing the ventral abdomen and back, were more frequently affected in the severe group than in the moderate group. These results demonstrate that diet-induced dermatitis is a disease of the entire body affecting all regions in different intensity (**Table 3**).

**Table 3:** Statistical differences between single regions by analyzing the frequency of the development of NASH dermatitis between the single judgment groups (Severe, moderate and mild NASH dermatitis).

	Moderate vs. Mild	Severe vs. Mild	Severe vs. Moderate
Region 1	0.086	**0.031**	0.831
Region 2	0.084	**0.007**	0.264
Region 3	**0.030**	**0.001**	0.137
Region 4	0.165	**0.026**	0.487
Region 5	0.281	**0.004**	**0.031**
Region 6	0.086	**<0.001**	**<0.001**
Region 7	0.333	**0.004**	**0.032**
Region 8	0.092	**0.006**	0.199

Bold values are statistically significant.

### 3.3 Diagnostic evaluation of the diet-associated dermatitis

The diagnostic evaluation considered three aspects: (1) Complete blood count and blood culture sampling. (2) Microbiological skin swab tests and (3) Histological evaluation of the NASH dermatitis lesions.

### 3.4 Complete blood count and clinical chemistry parameter

The inflammatory parameters measured in the complete blood count and CC parameter are shown in **Table 4**. There was no significant difference in all included animals during the course of the study.

**Table 4:** Inflammatory parameter of the complete blood count results taken prior and each second week during the NASH diet application.

	Severe	Moderate	Mild	p-value
WBC-W(-1) (10^9^/l)	14.1 ( ± 5.81)	16.8 ( ± 1.11)	16.8 ( ± 4.41)	0.601
LYM-W(-1) (10^9^/l)	7.84 ( ± 2.98)	8.37 ( ± 1.28)	9.42 ( ± 1.76)	0.651
MON-W(-1) 10^9^/l)	0.12 [0.11;0.14]	0.12 [0.09;0.14]	0.10 [0.10;0.15]	0.910
NEU-W(-1) 10^9^/l)	7.66 [3.43;8.27]	8.05 [7.91;8.47]	8.75 [6.49;8.84]	0.572
BUN-W(-1) (mg/dl)	9.80 ( ± 2.17)	7.75 ( ± 1.26)	8.00 ( ± 1.00)	0.197
TBIL-W(-1) (mg/dl)	0.30 [0.20;0.30]	0.30 [0.30;0.30]	0.30 [0.30;0.30]	0.210
ALT-W(-1) (U/L)	46.2 ( ± 25.4)	60.2 ( ± 5.74)	63.7 ( ± 14.6)	0.390
AST-W(-1) (U/L)	39.8 ( ± 20.1)	42.5 ( ± 5.20)	40.0 ( ± 6.56)	0.955
TP-W(-1) (g/dl)	4.60 [4.60;5.60]	4.65 [4.57;4.80]	4.80 [4.50;4.85]	0.947
WBC-W2 (10^9^/l)	19.1 ( ± 8.99)	15.8 ( ± 5.73)	17.9 ( ± 3.92)	0.793
LYM-W2 (10^9^/l)	9.45 ( ± 4.02)	8.94 ( ± 2.38)	11.7 ( ± 1.84)	0.512
MON-W2 (10^9^/l)	0.22 [0.12;0.25]	0.12 [0.10;0.13]	0.12 [0.11;0.14]	0.380
NEU-W2 (10^9^/l)	9.44 ( ± 5.24)	6.79 ( ± 3.90)	6.06 ( ± 2.12)	0.509
BUN-W2 (mg/dl)	11.6 ( ± 5.90)	13.5 ( ± 0.58)	13.0 ( ± 2.65)	0.781
TBIL-W2 (mg/dl)	0.30 [0.20;0.30]	0.30 [0.30;0.33]	0.30 [0.25;0.35]	0.660
ALT-W2 (U/L)	57.0 (5.29)	58.0 (9.93)	47.8 (16.1)	0.436
AST-W2 (U/L)	32.7 (3.51)	43.8 (13.8)	29.2 (9.34)	0.153
TP-W2 (g/dl)	6.64 ( ± 0.97)	6.35 ( ± 0.64)	6.00 ( ± 0.62)	0.565
WBC-W4 (10^9^/l)	16.2 ( ± 7.19)	21.4 ( ± 3.89)	18.9 ( ± 5.10)	0.442
LYM-W4 (10^9^/l)	8.57 ( ± 2.77)	10.8 ( ± 2.15)	10.4 ( ± 2.35)	0.394
MON-W4 (10^9^/l)	0.09 [0.09;0.14]	0.14 [0.11;0.17]	0.12 [0.11;0.16]	0.549
NEU-W4 (10^9^/l)	7.50 ( ± 4.59)	10.5 ( ± 5.25)	8.30 ( ± 3.45)	0.636
BUN-W4 (mg/dl)	10.6 ( ± 5.13)	15.0 ( ± 3.56)	13.3 ( ± 0.58)	0.298
TBIL-W4 (mg/dl)	0.30 [0.30;0.80]	0.45 [0.38;0.62]	0.30 [0.30;0.35]	0.575
ALT-W4 (U/L)	37.2 (14.8)	54.0 (5.48)	52.3 (10.3)	0.107
AST-W4 (U/L)	50.2 (36.9)	60.0 (15.6)	40.3 (7.77)	0.635
TP-W4 (g/dl)	6.02 ( ± 0.53)	6.30 ( ± 0.14)	6.30 ( ± 0.36)	0.513
WBC-W6 (10^9^/l)	21.4 ( ± 14.4)	20.1 ( ± 1.42)	22.1 ( ± 5.84)	0.963
LYM-W6 (10^9^/l)	8.92 ( ± 3.59)	9.08 ( ± 1.61)	9.51 ( ± 1.22)	0.954
MON-W6 (10^9^/l)	0.36 [0.15;0.36]	0.15 [0.12;0.17]	0.16 [0.15;0.17]	0.507
NEU-W6 (10^9^/l)	6.82 [4.85;12.9]	11.2 [9.28;12.9]	12.0 [9.83;14.9]	0.671
BUN-W6 (mg/dl)	10.2 ( ± 5.26)	14.0 ( ± 4.16)	14.0 ( ± 1.00)	0.359
TBIL-W6 (mg/dl)	0.62 ( ± 0.52)	1.57 ( ± 0.60)	0.53 ( ± 0.15)	0.030
ALT-W6 (U/L)	56.8 (13.2)	50.8 (14.3)	51.3 (8.14)	0.741
AST-W6 (U/L)	110 (71.4)	97.0 (21.4)	73.3 (22.5)	0.630
TP-W6 (g/dl)	6.50 [6.40;6.60]	6.40 [6.15;6.65]	6.40 [6.05;6.50]	0.848
WBC-W8 (10^9^/l)	19.9 ( ± 9.03)	15.7 ( ± 2.31)	20.0 ( ± 3.38)	0.659
LYM-W8 (10^9^/l)	9.82 ( ± 2.69)	7.13 ( ± 2.46)	8.60 ( ± 0.91)	0.435
MON-W8 (10^9^/l)	0.14 [0.11;0.14]	0.15 [0.12;0.46]	0.14 [0.12;0.15]	0.570
NEU-W8 (10^9^/l)	9.98 ( ± 6.29)	8.10 ( ± 2.50)	11.2 ( ± 4.27)	0.763
BUN-W8 (mg/dl)	9.50 ( ± 3.11)	14.0 ( ± 5.94)	14.0 ( ± 3.61)	0.329
TBIL-W8 (mg/dl)	0.50 [0.40;0.80]	0.70 [0.50;1.30]	0.60 [0.50;1.20]	0.652
ALT-W8 (U/L)	81.5 ( ± 19.6)	61.0 ( ± 9.27)	50.3 ( ± 4.04)	0.039
AST-W8 (U/L)	172 ( ± 90.0)	79.0 ( ± 23.0)	118 ( ± 25.1)	0.140
TP-W8 (g/dl)	6.50 [6.35;6.90]	6.30 [6.15;6.35]	3.70 [2.80;4.60]	0.096

WBC, weight blood count; LYM, Lymphocytes; MON, Monocytes; NEU, Neutrophils; BUN, Urea; TBIL, total Bilirubin; ALT, Alanine aminotransferase; AST, Aspartate aminotransferase; TP, total Protein.

### 3.5 Histological results

**Figure 4:**
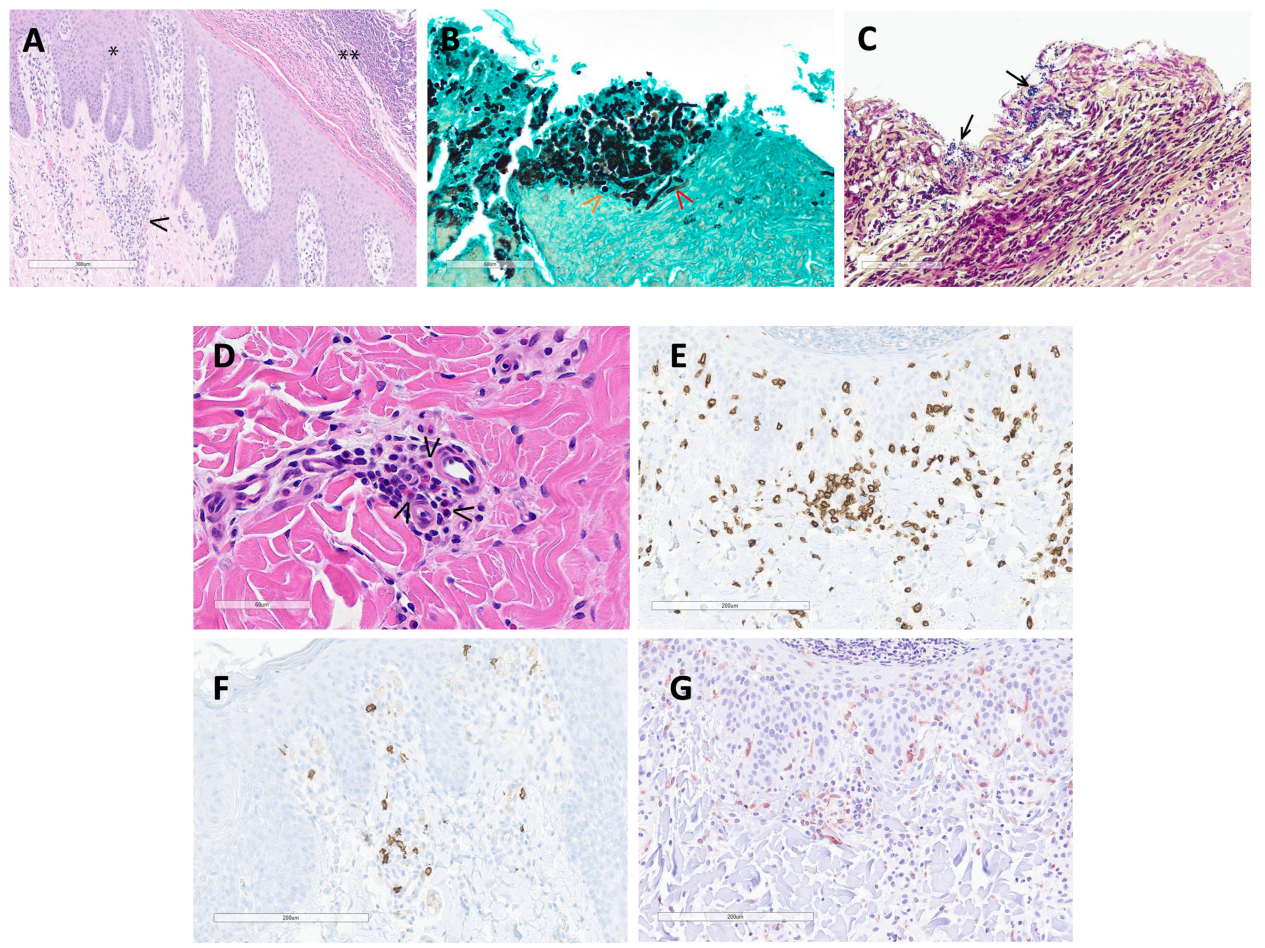
**(A)** (H&E 100X): The hyperplastic epidermis (*) is covered by a thick serocellular crust (**). The superficial dermis is expanded by edema and multifocally infiltrated by mixed inflammatory cells (macrophages, lymphocytes, eosinophils and neutrophils)(arrowhead) and scattered karyorrhectic debris. **(B)** (GMS stain 400X): Within the serocellular crust, there are numerous yeast (orange arrowhead) with fewer hyphal structures (red arrowhead) and scattered colonies of coccoid-shaped bacteria. **(C)** (Gram stain 400X): The gram stain highlights coccoid bacteria (arrows) within the serocellular crust. **(D)** (H&E 400X): In the superficial dermis, multifocal small caliber vessels are cuffed by eosinophils (arrowheads) and mononuclear cells. **(E)** (CD3 IHC 200X): Numerous T cells aggregate around small caliber vessels and are scattered within the superficial dermis and epidermis. **(F)** (CD20 IHC 200X): There are scattered B cells within the superficial dermis. **(G)** (Iba1 200X): Macrophages and dendritic cells are spread within the superficial dermis and epidermis.

Histopathologic interpretation of the skin punch biopsies was performed on three pigs. Frequently, the epidermis was covered by a thick serocellular crust containing lamellated eosinophilic material (keratin), degenerate and viable neutrophils, multifocal colonies of coccoid bacteria, and lightly basophilic spherules approximately 3-5 μm in diameter (**Figure 4A**). GMS stain highlighted yeast as well as scattered hyphal structures within the serocellular crust, most likely Candida sp. (**Figure 4B**). Gram stain identified gram-positive coccoid bacteria (**Figure 4C**). There was prominent acanthosis and slight spongiosis of the epidermis with multifocal anastomosing rete pegs and infiltration of the stratum corneum by numerous neutrophils. The superficial dermis was expanded by edema and infiltrated by a mixture of eosinophils, lymphocytes, macrophages, and occasional neutrophils surrounding multifocal small caliber vessels (**Figure 4D**). IHC for CD3 and CD20 identified numerous T cells, with fewer B cells, within the superficial dermis and frequently surrounding vessels (**Figures 4E, F**). IHC for Iba1 identified scattered macrophages, and possible dendritic cells, within the superficial dermis as well as the epidermis (**Figure 4G**). Evidence of a primary infectious dermatitis, such as dermatophytosis, was not observed in examined sections.

### 3.6 Microbiological skin swab test

To characterize the bacteria involved in the epidermitis, skin swabs were collected from two animals of the severe dermatitis group which had been on the NASH diet for 4 weeks. Bacterial culture identified in both animals *Staphylococcus aureus*, *Pseudomonas* sp., *Enterococcus faecalis* and *Klebsiella pneumonia* on the surface of the NASH skin lesions. The antibiogram of these germs are reported in **Table 5**. In addition to the detection of different bacterial species, *Candida tropicalis* was also isolated from skin lesions.

**Table 5:** Antibiogram of the four identified bacteria isolated from cutaneous swab tests with associated minimum inhibitory concentration (MIC) given in mcg/ml.

Antibiotics	*Staphylococcus aureus (MIC)*	*Pseudomonas* sp. (MIC)	*Enterococcus faecalis (MIC)*	*Klebsiella pneumoniae (MIC)*
Ampicillin	R (>16.0000)	R (>16.0000)	S (1.0000)	R (>16.0000)
Amoxicillin/Clavulanic acid	–	–	S	S
Ceftiofur	S (>2.0000)	R (8.0000)	R (>8.0000)	R (>8.0000)
Clindamycin	R (>16.0000)	NI (>16.0000)	R (>16.0000)	NI (>16.0000)
Danofloxacin	NI (>1.0000)	NI (0.5000)	NI (>1.0000)	NI (0.5000)
Enrofloxacin	R (>2.0000)	R (1.0000)	R (1.0000)	R (1.0000)
Florfenicol	I (4.0000)	R (>8.0000)	I (4.0000)	I (4.0000)
Gamithromycin	NI (>8.0000)	NI (>8.0000)	NI (<=1.0000)	NI (>8.0000)
Gentamicin	S (<=1.0000)	S (<=1.0000)	R (16.0000)	S (<=1.0000)
Neomycin	R (16.0000)	S (<=4.0000)	R (>32.0000)	S (<=4.0000)
Penicillin	R (>8.0000)	R (>8.0000)	S (2.0000)	R (>8.0000)
Sulfadimethoxine	R (>256.0000)	R (>256.0000)	R (>256.0000)	R (>256.0000)
Spectinomycin	NI (>64.0000)	NI (64.0000)	NI (64.0000)	NI (32.0000)
Tetracycline	R (>8.0000)	NI (4.0000)	R (>8.0000)	R (>8.0000)
Tiamulin	R (>32.0000)	R (>32.0000)	R (>32.0000)	R (>32.0000)
Tildipirosin	NI (8.0000)	NI (>16.0000)	NI (>16.0000)	NI (16.0000)
Tilmicosin	NI (<=2.0000)	NI (>16.0000)	NI (>16.0000)	NI (>16.0000)
Trimethoprim/Sulphamethoxazole	R (>2.0000)	R (>2.0000)	R (<=2.0000)	R (>2.0000)
Tulathromycin	NI (32.0000)	NI (>64.0000)	NI (<=8.0000)	NI (>64.0000)
Tylosin (Tartrate/Base)	NI (2.0000)	NI (>32.0000)	NI (4.0000)	NI (>32.0000)

Blood culture samples taken in W (-1) and W4 were negative for aerobic and anaerobic bacteria, excluding bacteremia in these animals.

### 3.7 Evaluation of treatment approaches for NASH dermatitis

**Figure 5:**
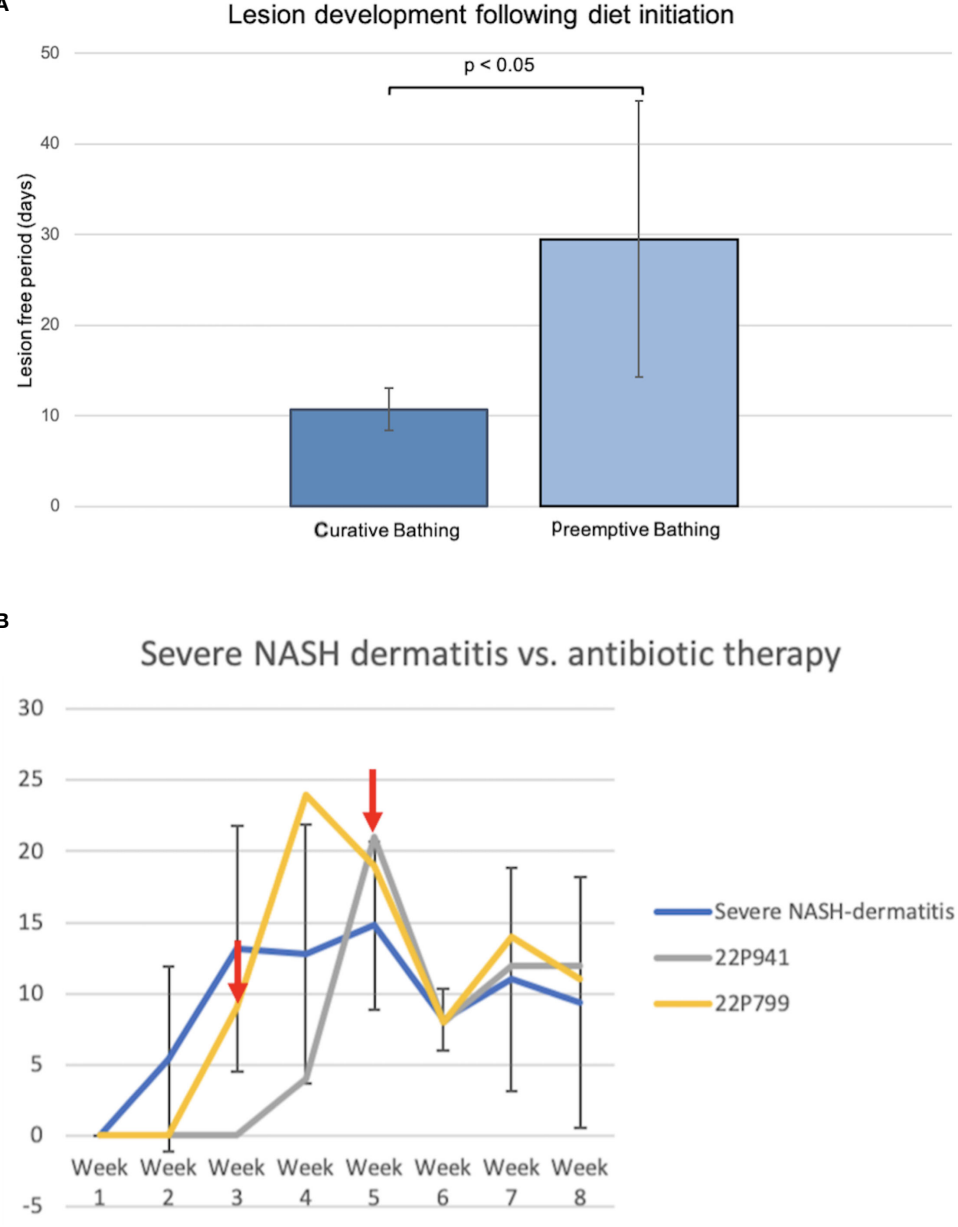
**(A)** Comparison of curative and preemptive bathing following the diet initiation. The curative approach is defined as up-to-daily topical bathing on veterinary discretion based on the lesion severity. The preemptive approach is defined as weekly bathing starting with the initiation of the diet application and independent of the presence of lesions. **(B)** Course of NASH dermatitis according to the developed skin judgment along with the 8-week NASH-diet feeding period. (Red arrow: start of the antibiotic treatment application in the individual pig).

Comparing both topical treatment approaches of the curative and preemptive setting indicated a potential benefit of preventive bathing starting with the initiation of the diet in pigs. The first four pigs fed the NASH diet developed dermatitis lesions approximately 10 days after the start of the diet. The first hypothesis for the development of the NASH dermatitis was based on the longtime physical contact of the pig skin with the NASH diet. To avoid this longtime skin contact and to reduce the diet-related local irritation of the skin, a local preemptive washing of the pigs by using 4% chlorohexidine shampoo was conducted in the following group of animals. The establishment of the preemptive bathing was associated with a significant delay in the onset of diet-associated dermatitis lesions (no bathing: 10.75 days ( ± 2.36), preemptive bathing: 29.5 days ( ± 15.22), p = 0.038) (**Figure 5A**).

Two animals of the severe NASH dermatitis group were treated with antibiotics due to a rapidly progressing dermatitis. In the first animal (22P799) of the severe group, enrofloxacin was empirically administered following the 3^rd^ week of diet application. However, a direct treatment effect of this therapy could not be observed, and the NASH dermatitis score was progressing in week 4 of the study course. Due to the progress of the dermatitis and based on culture and sensitivity results, the antibiotic treatment was switched to amoxicillin/clavulanic acid in week 4 of the study. This treatment led to a significant drop in the skin score from 19 points to 8 points.

Reported resistance in cultured organisms later in the study explained the initial failing response to enrofloxacin and confirmed the susceptibility of the cultured organisms to amoxicillin/clavulanic acid. Additionally, the positive treatment effect was confirmed during the clinical course of an additional pig (22P941) with severe NASH dermatitis. In this pig, the NASH dermatitis score decreased from 21 points (Week 5) to 8 points in the following week following the amoxicillin/clavulanic acid application (**Figure 5B**).

## 4 Discussion

This study demonstrates the first report of dermatitis in a large animal model of nonalcoholic steatohepatitis. Further, this manuscript characterizes the underlying etiology and treatment of dermatitis, demonstrating that multiple factors must be considered in the development of translational animal models, including factors critical to study design and unique to the selected species’ biology, husbandry, and welfare. The characterization of adverse effects of an animal model, including unexpected manifestations of study manipulations, is vital to preserving animal wellbeing and welfare. This knowledge is necessary for investigative staff and research partners directly or indirectly involved in the study, including veterinary staff and members of animal research committees, as knowledge of these adverse effects is essential for a comprehensive assessment of the project prior to and during the study. Due to the central role of the liver in systemic and metabolic processes, detailed knowledge of potential side effects is particularly essential in models of chronic liver disease.

Feeding a methionine- and choline-deficient, high fat diet is a frequently used method of inducing NASH. Extensive knowledge regarding the translatability, limitations, and adverse side effects of this model has been published but primarily covers rodents. Although rodent models are still widely used, this approach is increasingly being transferred to large animal models, such as swine. Published manuscripts describe the development and potential use of these swine NASH models in the context of translational research but do not discuss the side effects of disease induction in this species (Schumacher-Petersen et al., 2019; Pedersen et al., 2020; Herrera-Marcos et al., 2022).

The observed hypersensitivity reaction with secondary dermatitis in this study pigs raised multiple questions regarding etiology, animal welfare, required monitoring, and treatment. Pathologic and microbiological findings were reported with recommendations for monitoring and specific treatment options discussed. In addition to dermatitis, an impaired growth rate, steatorrhea, and anemia were also observed. To our knowledge, the presented data on the side effects of NASH induction in pigs, including evaluation of diet-associated dermatologic symptoms, represents the first characterization of the side effects of NASH induction in a large animal model.

All animals in the study developed a hypersensitivity reaction with secondary pustular dermatitis following NASH induction. While mouse models of NASH have demonstrated coat impacts, with fatty, shiny fur observed in animals on the diet (Kroh et al., 2020), neither research with rodents nor Gottingen or commercial pigs has demonstrated dermatitis nor other symptoms consistent with a hypersensitivity reaction (Schumacher-Petersen et al., 2019; Pedersen et al., 2020; Herrera-Marcos et al., 2022). This previously unreported dermatologic manifestation likely has a multifactorial cause with genetics, husbandry, and environmental conditions playing a role, but the potential impact of the NASH-induction diet must also be considered.

Amino acids play a key role in protein production, immunologic function, and tissue homeostasis. Methionine serves as a precursor for other amino acids (Martínez et al., 2017) and impacts the function of the skin via action on collagen production, immune system activation, and counteracting oxidative stress. Dietary methionine has been linked to direct dermatologic impact, with methionine supplementation linked to improved coat and skin characteristics and reduction of allergic dermatitis in domestic animals (Zhang et al., 2013). While choline’s role in the skin is less-well established, dietary enrichment with choline is associated with enhanced skin immune function and recession of skin lesions (Yuan et al., 2021). Given the functions of these key amino acids, the provision of a methionine- and choline-deficient diet could play a critical role in alteration of the structural and immunologic role of the skin, predisposing to the dermatologic lesions present in these animals. While the exact cause is unknown, the provided data regarding lesion progression, clinical monitoring, pathogenesis, and potential treatment approaches represents important background information for further investigation. 

A wealth of research connects multiple disease states to the gut microbiome, with far-reaching impacts on various body systems (Suez et al., 2014; Voigt et al., 2016; Zhao and Wang, 2020; Singh et al., 2022). While recent research has emphasized the vital role of the gut microbiome on various body systems, alterations in the commensal organisms of the skin have similarly been linked to the development of dermatologic disease. Insults to the skin from altered environmental conditions, parasites, or trauma can lead to dysregulation of the skin microbiome, overgrowth of pathogenic organisms, and secondary dermatitis (Swe et al., 2014). Microflora-induced disease is not limited to the activity of abnormal flora; these same predisposing factors can also cause skin commensals, which traditionally play a protective role, to proliferate and cause dermatitis. In addition to dermatitis caused by skin dermal site flora, well known from preclinical toxicity studies in Goettingen minpigs are fungal infections with *Candida* sp. causing therapy-resistant dermatitis (Ramot et al., 2017). Considering this knowledge and the microbiological results in this study, a hypothesis may be made that bacterial and fungal infections are ultimately causative of the observed dermatitis. However, in this case, the primary diet-induced hypersensitivity reaction in study animals in combination with husbandry and environmental factors likely led to an altered skin barrier, predisposing existing skin flora to overgrow and cause disease. By considering these findings, a variety of potential treatment approaches emerge in the context of large animal studies of chronic liver disease in pigs.

In the present study, dermatitis was adequately managed via bathing and targeted antibiotic therapy. Preemptive bathing of study animals prior to the development of the dermatitis showed positive effects on the time of initial lesion development, delaying average lesion development by nearly 20 days. Bathing the animals regularly limited the duration of dermatitis and avoided systemic treatment which could pose a risk to the diseased liver. While the bathing itself did not address the underlying hypersensitivity, it represented an opportunity to mitigate the disease. However, this treatment required considerable time and labor for staff, representing a limitation of this approach, particularly in studies involving larger animal numbers.

Treatment with targeted antibiotic therapy represented an alternate approach for managing severe dermatitis refractive to bathing. Antibiotic therapy successfully resolved the dermatitis in those two animals that failed to respond to bathing alone but required culture and sensitivity for appropriate antibiotic choice, delaying treatment initiation and representing a financial cost. Although both treatments evaluated here did not act on the underlying hypersensitivity reaction, they were effective at managing even severe animals through study endpoint. While systemic or local immunomodulatory agents such as prednisone would be first-line treatment in affected humans and companion animals, due to their far-reaching effects on physiology and the model of induced liver disease, their use was not evaluated (Zhao et al., 2016). The use of antibiotics in severely affected pigs was used to preserve animal welfare and ensure that affected animals could remain on study, but systemic oral antibiotic use has known effects of the intestinal microbiome of swine, influencing both microbial populations and gene expression (Looft et al., 2012). The effects of these alterations to the gut microbiome both to the diseased study liver as well as far-reaching physiological impacts requires further study. These findings and the data on microbiological colonization of the lesions may be useful in further studies to evaluate additional treatment options and improve the management of the hypersensitivity reaction in these animals.

Microbiological identification and antibiotic sensitivity testing were critical to evaluate the population of organisms on the skin of affected pigs and guide treatment. Skin culture of affected pigs highlighted skin commensals, rather than primary pathogens, as the underlying cause of the secondary infectious dermatitis. Culture results and historical knowledge of antibiotic resistance in the facility guided initial antibiotic therapy for severely affected animals, as the delay between sample collection and sensitivity results required initial empiric antibiotic treatment. Antibiotic treatment was then re-evaluated in the face of sensitivity results, guiding treatment decisions for that and subsequent affected animals. Given these findings, investigators managing suspect diet-induced dermatitis in swine models of NASH should consider empiric antibiotic therapy that targets common skin commensals but confirm the appropriateness of treatment via culture and sensitivity.

While the development of an unexpected dermatitis in study pigs was most remarkable, animals on study also had an impaired grow rate and developed steatorrhea and anemia, with variable similarities to previous rodent and swine models. Evaluation of weight logs for all animals on study demonstrated a static size, with impairment of weight gain and a lean body condition compared to normal feed pigs. While the weight loss and secondary welfare impact of the methionine- and choline-deficient nutritional NASH model in rodents is a well-established side effect with active research investigating the welfare impact of other nutritional NASH models, failure to gain weight has not been reported in other studies of NASH induction in pigs (Matsumoto et al., 2013; Kroh et al., 2020). Studies with Goettingen minipigs demonstrated variable weight gain as compared to normal chow control animals but all animals demonstrated weight gain over the feeding period. It may be inferred from these observations that pigs are more suitable than rodents for the induction of NASH not only in terms of their physiological characteristics but also in terms of better compensation of diet-associated impaired weight gain. However, the potential impairment of weight gain requires a close monitoring of study pigs, with regular assessment of body condition.

In contrast, the mild, non-symptomatic anemia in study animals had previously been demonstrated in Gottingen models of NASH and in rodents on choline-deficient diets, confirming this as an expected side effect of the model (Engel, 1948; Pedersen et al., 2020). While this side effects of NASH induction were less dramatic than the others, it is an important consideration when evaluating physiologic parameters of study animals.

Markedly important from a husbandry aspect was the ubiquitous steatorrhea in all study animals. This was expected given the high fat nature of the diet and was an important outcome for veterinary and animal husbandry staff, as this may have contributed to perceived deficiencies in weight gain. However, additional data is needed to further investigate this observation and to rule out confounding factors of the presented study.

Characterization of the adverse side effects of the methionine-choline deficient, high-fat diet was limited by low number of investigated animals. Additionally, all animals in the study were female, approximately the same age, with ten of twelve animals from the same supplier and genetic background. In addition to limited animal numbers and diversity, efforts to compare the severity of dermatitis between animals and evaluate treatment success were stymied by the lack of an established skin-scoring system in pigs. Dermatologic scoring systems are well-utilized in spontaneous and induced disease models in rodents, allowing for the evaluation of treatment efficacy and comparison of severity between animals, but are not established in swine (Hampton et al., 2012; Debes et al., 2020; Oh et al., 2021; Feng et al., 2022). While pigs are prone to develop numerous dermatologic diseases, primarily post-mortem scoring trials have been developed for the evaluation of carcass characteristics at slaughter. The lack of an approved evaluation system for swine dermatologic conditions combined with established rodent skin scoring systems gave rise to the preliminary gradation system described here (Hampton et al., 2012). However, this system for grading the skin is only based on a retrospective review of clinical records by a veterinarian involved in the study. Furthermore, the study was designed as a single -reviewer assessment to enable an exploratory judgment of the included animals. Without verification of the assessment criteria in a prospective controlled study, the used skin judgement criteria are of limited use for real-time clinical assessment. Additional research is needed to evaluate intra- and inter-scorer reliability and assess the correlation between severity scoring and veterinary intervention. Additionally, further diagnostic testing, including expansion of culture and sensitivities and histopathologic sampling, including further characterization of the suspect hypersensitivity reaction, would provide a more complete description of dermatitis development. However, the study was able to identify adverse side effects of a methionine-choline deficient, high fat diet for inducing NASH in pigs. Outside of the dermatitis, these effects are not unexpected in the context of diet-induced NASH models in rodents and large animals but require adequate management from all study members. The diet-associated dermatitis, if managed pre-emptively and aggressively, Does not pose a threat to the animal’s welfare and does not justify the rejection or premature termination of such a study in large animals.

## 5 Conclusion

Gottingen and domestic pigs fed a methionine-choline deficient, high fat diet to induce NASH developed unexpected side effects of disease induction, most notably a hypersensitivity reaction with secondary dermatitis, failure to gain weight, steatorrhea, and anemia. This is the first report of dermatologic manifestations in rodents or pigs with diet-induced NASH and demonstrates the importance of commensal microflora in animal models of disease. With pre-emptive knowledge of these side effects and potential treatment avenues, study pigs can be appropriately managed through NASH induction and serve as valuable translational models.

## Data availability statement

The raw data supporting the conclusions of this article will be made available by the authors, without undue reservation.

## Ethics statement

The animal study was approved by Institutional Animal Care and Use Committee (IACUC) of Mayo Clinic. The study was conducted in accordance with the local legislation and institutional requirements.

## Author contributions

PF: Conceptualization, Formal analysis, Methodology, Writing – original draft, Writing – review & editing. JL: Data curation, Formal analysis, Investigation, Project administration, Visualization, Writing – original draft. SH: Investigation, Visualization, Writing – review & editing. JJ: Formal analysis, Visualization, Writing – review & editing. BPA: Supervision, Writing – review & editing. LF: Data curation, Validation, Writing – review & editing. AM: Data curation, Project administration, Writing – review & editing. AS: Project administration, Writing – review & editing. BA: Project administration, Writing – review & editing. MR: Data curation, Formal analysis, Methodology, Validation, Writing – review & editing. SN: Conceptualization, Funding acquisition, Project administration, Supervision, Writing – review & editing.
