# Supplementary material for: Clinical characterization of a hypersensitivity mixed bacterial and fungal dermatitis in a translational model of porcine NASH
Source: Front Cell Infect Microbiol. 2024 Jan 24;13:1277045. doi: 10.3389/fcimb.2023.1277045 (PMC10847572; doi:10.3389/fcimb.2023.1277045)
Supplement: Supplementary file 1 [file DataSheet_1.pdf]

Supplementary Material:

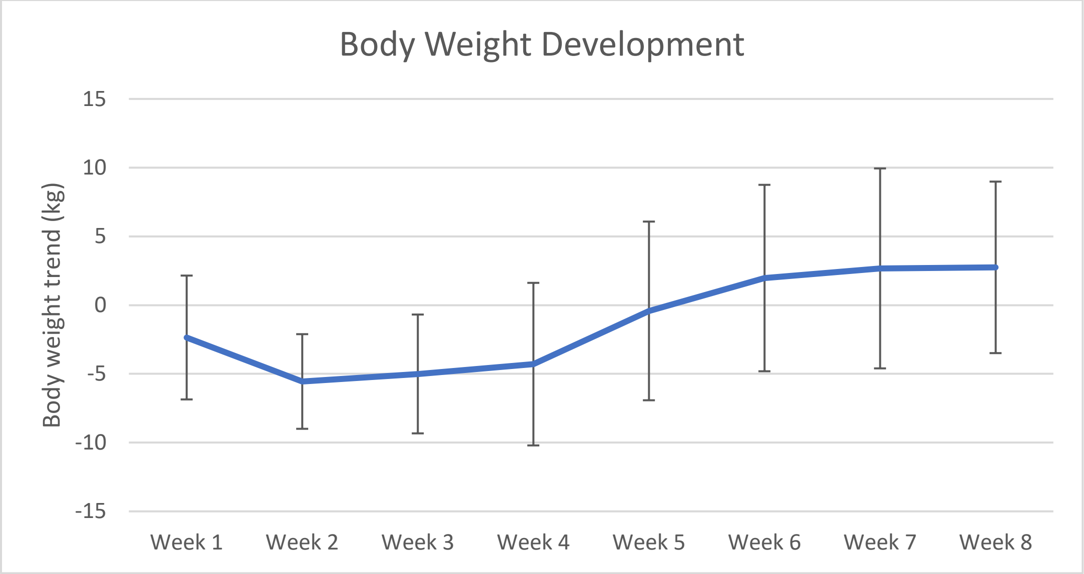

Supplement Fig 1: Average weekly trend in body weight of the 10 domestic pigs over the study course. Average bodyweight at each timepoint post-diet initiation was compared to body weight collected prior to diet start.

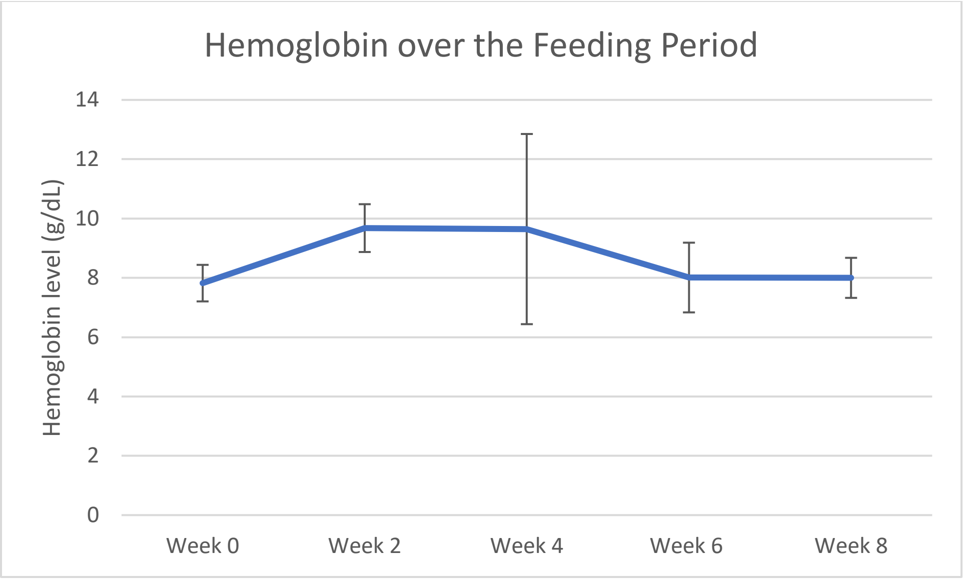

Supplement Fig 2: Average hemoglobin values of the 10 domestic study pigs over the study course. Blood samples were collected for hemoglobin evaluation every two weeks. Hemoglobin values below 11 g/dL were indicative of mild anemia.

Animal #	Strain	Starting Weight (kg)	Final Weight (kg)	Max Skin Score	Washing interval	Antibiotic	Diagnostics
1	Domestic pig	14	18.8	15	7x /week		
2	Domestic pig	13	13.6	22	7x/week		
3	Goettingen	5.6	9.6	18	7x/week		
4	Goettingen	6.0	8	21	7x/week		
5	Domestic pig	13.5	14.8	2	1x/week		
6	Domestic pig	12.5	15.2	0	1x/week		
7	Domestic pig	11.5	13	12	3x/week		
8	Domestic pig	11	13	24	7x/week	Enrofloxacin 7.5 mg/kg, once daily x 7days Amoxicillin/Clavulanate 20 mg/kg twice daily x 20 days	C&S
9	Domestic pig	10.5	11.2	16	7x/week	Amoxicillin/Clavulanate 20 mg/kg twice daily x 13 days	C&S, Skin biopsy for histopathology
10	Domestic pig	11	13.5	13	5x/week		C&S, Skin biopsy for histopathology
11	Domestic pig	11.5	13.2	6	3x/week		Blood Culture
12	Domestic pig	12.5	14.2	13	3x/week		C&S, Skin biopsy for histopathology, Blood Culture

Supplement Fig 3: Panel of all included animals.
